# The Polish Version of the Nursing Delirium Screening Scale (NuDESC PL)-Experience of Using in Nursing Practice in Cardiac Surgery Intensive Care Unit

**DOI:** 10.3390/ijerph181910108

**Published:** 2021-09-26

**Authors:** Sabina Krupa, Ozga Dorota, Adriano Friganovic, Wioletta Mędrzycka-Dąbrowska, Krzysztof Jurek

**Affiliations:** 1 Institute of Health Sciences, College of Medical Sciences of the University of Rzeszow, Poland St. Warzywna 1A, 35-310 Rzeszow, Poland; sabinakrupa@o2.pl (S.K.); gdozga@poczta.fm (O.D.); 2 Department of Anesthesiology and Intensive Medicine, University Hospital Centre Zagreb, 10000 Zagreb, Croatia; adriano@hdmsarist.hr; 3 Department of Nursing, University of Applied Health Sciences, Mlinarska Cesta 38, 10000 Zagreb, Croatia; 4 Department of Anaesthesiology Nursing & Intensive Care, Faculty of Health Sciences, Medical University of Gdansk, 80-211 Gdansk, Poland; 5 The Institute of Sociological Sciences, The John Paul II Catholic University of Lublin, 20-950 Lublin, Poland; kjurek@interia.eu

**Keywords:** nursing delirium screening scale, disorientation, delirium, screening

## Abstract

Introduction: Delirium is a common complication of patients hospitalized in Intensive care units (ICU). The risk of delirium is estimated at approximately 80% in intensive care units. In the case of cardiac surgery ICU, the risk of delirium increases due to the type of procedures performed with the use of extracorporeal circulation. The aim of this study was to provide an official translation and evaluation of Nursing Delirium Screening Scale (NuDESC) into Polish. The NuDESC scale is a scale used by nurses around the world to detect delirium at an early stage in treatment. Methods: The method used in the study was the NuDESC tool, which was translated into Polish. The study was conducted by Cardiac ICU nurses during day shift (at 8 a.m.), night shift (at 8 p.m.) and in other situations where the patients showed delirium-like symptoms. Results: Statistically significant differences were observed between the first and second day in the studied group of patients in the case of illusions/hallucinations. Delirium occurred more frequently during the night, but statistical significance was demonstrated for both daytime and nighttime shifts. It was not demonstrated in relation to the NuDESC scale in the case of insomnia disorders. The diagnosis of delirium and disorientation was the most common diagnosis observed in patients on the first day of their stay in the ICU, followed by problems with communication. Delirium occurred on the first day, mainly at night. On the second day, delirium was much less frequent during the night; the biggest problem was disorientation and problems with communication. Conclusion: This study contributed to the development of the Polish version of the scale (NuDESC PL) which is now used as the Polish screening tool for delirium detection. The availability of an easy-to-use nurse-based delirium instrument is a prerequisite for widespread implementation.

## 1. Introduction

Delirium is a problem that affects an increasing number of hospitalized patients. Despite many studies and available tools it is often undiagnosed, which increases the morbidity rate in the study population. Delirium is a cognitive disorder that affects 3–29% of hospitalized patients [1]. There are some tools in the literature that assess delirium. Most delirium assessment tools are dedicated only to doctors. The psychometric properties of NuDESC were determined using the categories of the Confusion Evaluation (CAM) performed by nurses and psychiatrists [1]. The Nursing Delirium (NuDESC) screening scale is a five-point scale. The fifth item evaluates atypical psychomotor delay, taking into account the medical condition (reaction delay, little or no spontaneous actions/words; for example, when the patient is pushed, the reaction is delayed and/or the patient is unable to wake up) [1]. There are no conventional nursing delirium screening tools for ICU patients. Effective early diagnosis of delirium has been recognized as one of the first priorities in the approach to the patient in the ICU [2]. The above-mentioned delirium screening scale has not been officially translated into Polish or validated in Poland. The need to equip ICUs with dedicated monitoring tools is obvious, as is the understanding that early identification of delirium guarantees early and appropriate treatment. NuDESC was initially developed by specialists in internal medicine and oncology, and then also applied to ICUs, but so far has not been translated or validated in the Polish language. First authors evaluated two delirium screening tools for detecting post-operative delirium in the elderly [3]. Post-operative delirium is the best known form of post-operative cognitive impairment in all patient groups, especially in the elderly. Delirium is a syndrome with serious consequences, increasing mortality, the severity of the patient’s condition and the length of hospital stay. Nursing staff spend most of their time with the patient in ICU, which has been repeatedly confirmed by tests [4,5,6,7]. Research shows that workload is increasing with the worsening COVID-19 pandemic [8].

The aim of this study was to provide a translation of NuDESC into Polish in accordance with the guidelines of the International Society for Pharmacoecomonics and Outcome Research (ISPOR). The translation process followed the guidelines of the Translation and Cultural Adaptation Group (TCA Group) [9]. The authors decided to make and use the translated tool in the conditions of advanced nursing practice, with a simultaneous attempt to correlate it with variables.

## 2. Methods

### 2.1. Study Design

A prospective repeated-measure descriptive study was conducted.

### 2.2. Sample

The method used in the study was the NuDESC tool, which was translated into Polish. The NuDESC scale is a scale used by nurses around the world to detect delirium at an early stage in treatment. The study was conducted by Cardiac ICU nurses during day shift (at 8 a.m.), night shift (at 8 p.m.), and in other situations where the patient showed symptoms similar to delirium.

### 2.3. Nursing Delirium-Screening Scale-NuDESC

The NuDESC is an observational screen for delirium that assesses five items: (1) disorientation, (2) inappropriate behavior, (3) inappropriate communication, (4) hallucination, and (5) psychomotor retardation. Each characteristic is scored by severity from 0 (absent) to 2 (severe) (a table with the analysis of activities on the NuDESC scale is included in the results). The evaluation is based on a composite of observations collected over a period of 12 h. It takes less than 2 min to complete and is designed for use by nursing staff. A threshold of ≥2 is considered a positive screen for delirium [1]. The NuDESC was administered by trained nurses in participating hospital units. All nurses were given an in-service regarding use of the NuDESC. A nurse champion on each floor provided continuing education and encouraged compliance with NuDESC administration, which averaged 71%. The highest NuDESC score recorded in the electronic medical record over the two-day evaluation period and the 12 h immediately preceding it (five observations) was used and compared to the reference standard. The scale was not used during the entire stay of patients at the hospital, because it was a group of ICU patients after cardiac surgery, after which they returned to their original department. 

Our main research was preceded by a pilot study. Three independent research nurses conducted research on a group of 30 patients hospitalized in the Intensive care unit (ICU). Nurses assessed the same patients. Cohen’s kappa coefficient (interrater reliability) of NuDESC scores between research nurses was 0.78 in the pilot study. The degree of agreement among independent observers was high.

### 2.4. Setting and Procedure

The guidelines of the International Society for Pharmacoecomonics and Outcome Research (ISPOR) were followed [9] to obtain a repeatable translation process. The translation procedure had been defined in advance, along with all steps of the translation and evaluation process. The translation team defined the exact timeframe and administrative aspects and performed the translation and evaluation of the tool. It analyzed publications in the field of delirium in the ICU. Upon approval, the scale was translated into Polish. The Polish version of the scale was used in a study of patients in the Intensive Care Department in south-eastern Poland. The study lasted 13 months in 2020. The research was carried out by nurses who had previously undergone a training process on how to mark subsequent scale elements. Patients received the consent questionnaire on admission to the ward. Potential respondents were selected randomly, in accordance with their duties and possibilities at the time. Before cardiac surgery, the respondents received information about the possible occurrence of delirium after the procedure and agreed to use the scale. The study included 223 patients, but 21 questionnaires were eliminated during the study, which constitutes 9.41% (the studies were not conducted during the hours specified by the scale). Ultimately, 202 patients after cardiac surgery were included in the study, or 90.58% of the cohort (File S1). Nurses interested in participating in the study received full information about purpose, the principles of data collection, and the possibility of withdrawing patients from the survey at any stage, and ensured the anonymity and confidentiality of the data. The study was conducted by 41 nurses from the Cardiac ICU (all nurses from the ward). Each nurse agreed to conduct research and underwent training in this field. Nurses completed the examinations at designated times of their day and night shift. NuDESC was completed on each nursing shift. If delirium was suspected, nurses, in consultation with doctors, selected pharmacological treatment individually according to the patient’s condition. There is no specific protocol for the use of pharmacotherapy in the Cardiac ICU due to the varied status of patients.

### 2.5. Ethical Considerations

This study was approved by the Bioethics Commission of the University of Rzeszów (KBE No. 3/09/2017) and permission from the management of the hospital was obtained. The authors followed the guidelines of the Declaration of Helsinki (World Medical Association, 2013). After participation was stated on a voluntary basis, written and oral informed consent of the participants was obtained. Before the research process, permission was obtained from the researchers who developed the scale for the use of the Nu-DESC and the study of the validity and reliability of the scale for Polish society. Health workers in the clinics included in this study were informed about the purpose, content, and method of this study.

### 2.6. The Translation Process

Before starting the study in Poland, it was necessary to translate of the scale to Polish conditions. The author of the original version of the scale was informed about the translation and the author’s consent for the translation into Polish was obtained. 

#### 2.6.1. Forward Translation

After the initial division of tasks, the authors of the study prepared the material and submitted it for professional translation by a medical language translator. Two separate translations, independent of each other, were made to check the correctness of the translated scale. The finalized version was proofread by two of the translators.

#### 2.6.2. Reconciliation

The translations were then merged into one preliminary version. The Polish version was adapted to a clinical setting without changing the meaning. Each phrase was discussed carefully and compared between the four translations to find the most accurate and fitting phrase. The final wording was consented to when all translators agreed unanimously. Expressions like “orient to current reality” were those that were thoroughly covered by the team of authors. In the end, they used this expression without changing its meaning. 

#### 2.6.3. Back Translation

The preliminary version was then translated back into English by an experienced and certified language teacher without knowledge of the original English version. 

#### 2.6.4. Back Translation Review

The back translation of the preliminary Polish version was then thoroughly compared with the original text regarding the necessity of performing adjustments. This back translation showed no substantial deviations from the original after close comparison and assessment performed by the translating authors. The translation was done by an independent translator (lector, Center for Foreign Languages, University of Rzeszów, specializing in medical translation), who accepted the version sent by the research team. 

#### 2.6.5. Cognitive Debriefing

The authorized Polish version of the scale was assessed by the employees of the ICU Cardiac Surgery department. The evaluation team consisted of 38 nurses and four doctors (two anaesthesiologists and two cardiac surgeons). The NuDESC PL assessment performed by two professional groups and two different specialties did not reveal any basic difficulties in understanding the content or language. The evaluation also revealed that all elements can be easily used. There were no significant differences between the ratings of the nurses and doctors. 100% of the professionals considered the scale as being as understandable and feasible as the original version, which was attached to the Polish version.

#### 2.6.6. Final Report

Two of the authors reviewed the result as well as the whole process of translation. After the final positive evaluation and due to the good results of the cognitive debriefing, the authors approved the Polish version of the Nu-DESC. The final and approved version of NuDESC PL is now ready for clinical and scientific research (File S2). 

### 2.7. Statistics

Two groups were compared (days 1–2) with Student’s *t* test for dependent groups. This test is used when measuring an examined variable twice. To determine the correlation between quantitative variables, we was used the Pearson product–moment correlation coefficient, which is used to study the linear relationship between two features. We was used the McNemar test to determine the relationship between the variables measured on the qualitative scale; this test is used to verify the hypothesis of correspondence between the results of double measurements (the feature has two categories). For improved understanding the results of this study are tabulated to improve informed care decision-making [10].

### 2.8. Other Scales and Parameters Used in the Study

#### 2.8.1. Numerical Rating Scale (NRS)

A numerical scale was used to monitor the effectiveness of analgesic treatment (NRS—Numerical Rating Scale) on which the severity of pain is given by numbers from 0 to 10. It is more understandable than other scales and can also be used by people with visual impairments. A caregiver asks the patient to describe the severity of the pain by indicating the appropriate number, with 0 being no pain and 10 being the worst pain imaginable. For patients with acute pain, this type of examination is advisable; it can be repeated many times and provides useful data [11]. 

#### 2.8.2. Central Venous Pressure (CVP)

Central venous pressure (CVP) is the pressure in the thoracic vena cava near the right atrium. CVP is an important factor in critical care medicine because it can be used to estimate a patient’s fluid volume status, assess cardiac function, and gauge how well the right ventricle of the heart is functioning [12]. 

#### 2.8.3. Level of Sodium and Potassium in Blood of Cardiac Surgery Patients

The literature gives the correct concentration of potassium as between 3.5–5 (5.5) mmol/L. The sodium concentration in normal conditions is 138–145 mmol/L [12]. Electrolyte disturbances often accompany patients, especially those who are hospitalized in highly specialized departments. It is important to conduct research that shows the correlation between the correct level of sodium and potassium and the impact on the functioning of the body in the course of specific diseases [13].

#### 2.8.4. Insomnia Severity Index

Insomnia is a subjective disorder; Morin [14] developed the Insomnia Severity Index (ISI) to provide a brief self-report tool useful for assessing people’s subjective perceptions of sleep problems in different populations. Due to its brevity (seven items, max. 2 min per filling and 1 min per score), the ISI can be used as a screening measure in clinical practice, allowing the assessment of changes after treatment; it is also useful in epidemiological studies. The ISI results allow a clinical evaluation of the symptoms of insomnia. The ISI covers both the night and day components of insomnia, and measures (a) the subjective symptoms and consequences of insomnia and (b) the fears arising from these difficulties. Part of the ISI considers the diagnostic criteria for insomnia as formulated in the DSM-IV (Diagnostic and Statistical Manual of Mental Disorders). The instrument consists of seven items on the severity of falling asleep (first point) and difficulty in maintaining sleep (including waking up at night and early morning, points two and three, respectively), current sleep satisfaction (fourth point), interference with sleep difficulties on daily functioning (fifth point), visibility of impairment due to sleep disturbance (sixth point), and restlessness or anxiety attributable to sleep disorders (seventh point). All items have a 5-point Likert scale from 0 (not at all) to 4 (extreme), with a reference period of the previous 2 weeks. ISI exists in three different versions: self-administered (patient), administered by a significant other (e.g., spouse, parent), and administered by a physician. ISI scores range from 0 to 28 and can be interpreted as follows: 0–7 = no clinically significant insomnia, 8–14 = subliminal insomnia, 15–21 = clinical insomnia (moderate severity) and 22–28 = clinical (severe) insomnia. The ISI has been used in clinical research and practice for nearly 30 years. 

## 3. Results

### 3.1. Clinical Characteristics of the Respondent

As some patients left the ICU on the first and second day of their stay, the number of patients on the first and second day of stay was different. 112 men (55.4%) and 90 women (44.5%) participated in the study. The mean weight of the study population was 75.2 kg, with an average height of 168.9 cm and BMI of 26.2. The average stay for patients at the ICU was 54 days. It should be emphasized that patients remain in the CICU for two days after cardiac surgery, but in case of complications, such stay is extended. The mean stay of patients, both planned and complicated, with the CICU was 54 days. Statistical significance was demonstrated for the age and duration of patients’ stay in the ICU (night duty on the first day), as well as for the duration of patients’ stays in other situations (second day). During the first day of stay, during the day and night shift, most of the patients were above 50 years old (78.8%). The majority of patients (78.9%) were women. Over 80% of patients (80.8%) were overweight. In the second day of their stay, on day and night duty, patients were 50 years old (58.8%). More patients were women (60%) and more than half were of normal weight (54.4%).

#### Other Variables Related to the Study Population

In the study population, statistical significance in relation to pain was observed on both days. During the first day of hospital stay, the patients experienced the greatest level of pain at 10 p.m. (72.3%), and the lowest level at 3 p.m. (30.2%). On the second day of stay at the ICU, the patients experienced the greatest pain by 6 p.m. (55.4%) and the least at 2 a.m. and 6 a.m. (42.1%). The highest percentage of people with normal CVP in the first day of stay at the ICU was recorded at 8 a.m. and 9 a.m. (67.8%), and the lowest at 10 p.m., 2 a.m., and 6 a.m. (37.6%). On day 2, the highest percentage of people with normal CVP was recorded at 8 a.m. (23.3%) and the smallest at 12 a.m., 1 p.m. and 3 p.m. (14.4%). The statistical analysis showed a statistical significance for each hour and day of the patient’s stay in the ward. Ten and above were assumed to be correct CVP values. The sodium level in the following hours and days was analyzed on the ward in the study group. We recognized 135–145 mmol/L as a normal level of sodium. The highest percentage of patients with normal sodium levels was recorded at 3 p.m. The statistical analysis showed statistical significance at each hour and day of the patient’s stay at the ICU. The level of potassium in the hours and days following the patient’s stay in the ward was analyzed in the study group. As a normal level of potassium, we recognized 3.5–4.5 mmol/L. The level of five or greater was taken as the value considered inflammation. On the first day of patients’ stay in the ICU, the highest percentage of inflammation was recorded at 6 p.m. and 12 p.m. (88.1%). The lowest number of patients with developed inflammation was recorded at 6 a.m. (58.9%). In the case of the second day stay, inflammation was noted at 6 p.m. and 12 p.m. in 76.2%. The lowest number of patients with developed inflammation on day 2 of the stay was at 6 a.m. (53.5%). The analgesic management of patients after cardiac surgery should be adjusted to the intensity of the pain currently experienced by the patient. For practical purposes, the following pain ranges should be adopted using the NRS: mild pain: 1–4 on the NRS, moderate pain: 5–6 on the NRS, severe pain: 7 and above on the NRS [14]. The analysis included patients who assessed pain of moderate intensity (Table 1).

As indicated, the study also used the Insomnia Severity Index scale. This scale describes insomnia disorders in the studied population. Statistical significance was not demonstrated for the Insomnia Severity Index relative to the NuDesc scale (Table 2).

### 3.2. Delirium and Preliminary Analysis according to NuDesc PL

In the first 24 h after surgery, delirium occurred mainly at night. The maximum number of points received by the patients was 8. The most common problems of patients in the first day after surgery include disorientation, communication problems and psychomotor retardation. According to the scale, delirium occurred in the case of receiving 2 or more points. During the first day of stay at the ICU, 49 patients (24.3%) showed symptoms of delirium. During the night of the first day of stay, 136 patients (67.3%) had delirium. When the nurses noticed that a patient developed delirium, the NuDesc PL study showed that 10 patients (5%) had confirmed delirium. During the second day of stay at the ICU, 89 patients (44.1%) showed signs of delirium. During the night of the second day of stay, 84 patients (41.6%) had delirium. The nurses’ survey showed that 21 patients (10.4%) had confirmed delirium (Table 3).

We showed a detailed analysis of the intervention on the first and second days. Most of the interventions were performed with patients on the first day of stay during the day shift (8 a.m.). More patients needed intervention during the day, including when recalled to reality (68.8%), and for proper use of glasses and hearing aids (80.2%). More frequent interventions during the night (8 p.m.) included sleep support (76.2%). Interventions performed in patients were more often performed during the second day of stay at the ICU, during the day. This was influenced by the condition of patients; patients on the second day of stay are mostly stable patients, not requiring as much attention as on day 0. More patients needed intervention during the day, including when recalled to reality (69.8%), and for proper use of glasses and hearing aids (80.2%). More frequent interventions during the night (8 p.m.) included sleep support (73.8%) (File S3). 

### 3.3. Characteristics of the Most Common Problems of Patients Diagnosed with Delirium

Taking into account the diagnosis of delirium, disorientation was most often observed in patients on the first day, followed by communication problems, with delirium occurring on the first day mainly occurring at night. There was no delirium on the first day or in other situations assessed during shifts. In the case of the second day, delirium was much less frequent at night; however, the biggest problem remained disorientation and communication problems (Table 3). Table 3 shows the difference in the occurrence of delirium during days 1 and 2. On the second day, 44.1% of patients manifested delirium, and on the first day, 24.3%. Taking into account night duty on the first day, it can be seen that 67.3% of patients had delirium, and 41.6% on the second day. This may have been related to the first night of the ICU and the influence of external stimuli which are predictors of delirium.

Statistically significant differences were observed between first and second day in the studied group of patients in the case of illusions/hallucinations. Delirium occurred more frequently during the night, but statistical significance was demonstrated for both daytime and nighttime shifts (Table 4 and Table 5).

## 4. Discussion

This prospective study involving 202 patients has shown that the NuDESC can be used as a screening tool for assessment by nurses in Poland in routine clinical practice at a Cardiac ICU. This study is the first official translation of this tool into Polish in Poland. The NuDESC adaptation process took 13 months. Researchers had contact with the author of the original scales. Through the ISPOR process, the scale was translated and thus the concepts of the original instrument were preserved [9,15]. As with the Danish version of the scale, there were no differences in the assessment of the tool by nurses and doctors in our study [16]. According to scientific reports, delirium is one of the main symptoms seen in patients hospitalized in the intensive care unit [17,18,19]. This information was confirmed during our own research. The importance of this tool is in that not only doctors are able to diagnose delirium with it, but also nurses. Thanks to this, it is possible to detect delirium-related disorders even faster, because nurses stay for a longer time at the patient’s bedside [16]. In our own research, the nurses also diagnosed delirium. Delirium can take many forms and therefore changes throughout the day. Through the daily routine, certain elements of delirium may go unnoticed or be confused with other ailments. Late detection of delirium is associated with the progression of this phenomenon and with consequences at various levels of care and treatment [20,21]. The European Society of Anesthesiology (ESA) recommends using a validated delirium score for postoperative delirium screening (for example, CAM-ICU and Nursing Delirium Screening Scale) [22]. Through these recommendations, we decided to prepare the scales to work in Polish conditions. According to research by Turkish scientists, the Nu-DESC scale brings great benefits to patients after surgery. They indicated that this tool is fast and sensitive in terms of delirium diagnosis [21]. According to the study of validation values, in Turkey, as in other countries, the scale is sensitive enough to be used in everyday practice [23,24,25]. Such information is very relevant to nursing research that assesses and relates to the impact of application-oriented education [26]. The authors’ own research showed statistical significance in relation to insomnia disorders. Insomnia in the study by Lewandowska et al., is touted as one of the predictors for delirium in ICU patients [27]. A similar situation occurs with respect to pain. Pain and delirium are common in people with dementia or who have had surgery. In this publication, the authors present the problem of pain that positively correlates with the occurrence of delirium. Feast et al., describe pain occurring in 33% of people remaining in hypoactive delirium, and 56% in agitated patients [28]. 

## 5. Practical Implications

NuDESC is a delirium screening tool that can be combined with nursing care and practice. Future research into therapeutic interventions may benefit from the use of new delirium scales, including NuDESC. Knowledge of tools for delirium assessment can significantly improve the quality of care for patients hospitalized in the ICU. According to the results of this study, the symptoms of delirium, such as problems with communication, and illusions/hallucinations, occurred most often at night on the first day of the stay. The pain was also more intense in the first 24 h in the evening hours. Delirium on the first day was diagnosed in 67.3% of the respondents. The presence of delirium can be associated with pain, as described the literature. Consideration should be given to the need to use painkillers, especially at this time.

## 6. Study Limitations

In our analyses, the groups differ depending on the length of the patient’s stay in the ward. One department was included in the translation process, and an attempt was made to adapt the research tool to Polish conditions. NuDESC should be compared with the diagnosis of delirium according to the criteria of the Diagnostic and Statistical Manual of Mental Disorders-5 (DSM-5) with the NuDESC sensitivity and specificity calculation; therefore, in our study it was not possible to confirm the diagnosis of delirium. 

## 7. Conclusions

The aim of this study was to provide an official translation and evaluation of the Nursing Delirium Screening Scale (NuDESC) into Polish. With NuDESC PL, we provide an official Polish instrument for delirium screening which can detect all psychomotor types of delirium. NuDESC PL has the potential to be an instrument for screening delirium in Poland. According to the research results, the translation of the tool is correct, understandable for the respondents and can be used in Poland.

## Figures and Tables

**Table 1:** Pain level in the study group.

NRS	Pain Requiring Intervention Is a Number of Five or More				*p*
	First Day		Second Day		
	N	%	N	%	
NRS 8:00	65	32.2	93	46.0	0.007
NRS 12:00	94	46.5	93	46.0	1.000
NRS 15:00	61	30.2	91	45,0	0.003
NRS 18:00	75	37.1	112	55.4	<0.001
NRS 22:00	146	72.3	86	42.6	<0.001
NRS 2:00	135	66.8	85	42.1	<0.001
NRS 6:00	134	66.3	85	42.1	<0.001

NRS = Numerical Rating Scale; N = number of patients; *p* = significance level.

**Table 2:** Relationships between the occurrence of delirium and the Insomnia Severity Index.

NuDesc	ISI First Day		ISI Second Day	
	R	*p*	R	*p*
Day	−0.060	0.393	0.006	0.927
Night	0.001	0.990	−0.052	0.463
Other	−0.051	0.475	−0.024	0.729

*p* = significance level; R = correlation; D = day; N = night.

**Table 3:** Results of NuDesc PL during first day and during second in the ICU.

**Points**	**First Day**		**First Night**		**Second Other**	
	**N**	**%**	**N**	**%**	**N**	**%**
**Nu-DESC negative (<2)**	153	75.7	66	32.7	192	95.0
**Nu-DESC positive (≥2)**	49	24.3	136	67.3	10	5.0
**All**	202	100.0	202	100.0	202	100.0
**Points**	**Second Day**		**Second Night**		**Second Other**	
	**N**	**%**	**N**	**%**	**N**	**%**
**Nu-DESC negative (<2)**	113	55.9	118	58.4	181	89.6
**Nu-DESC positive (≥2)**	89	44.1	84	41.6	21	10.4
**All**	202	100.0	202	100.0	202	100.0

N = number of patients, Nu-DESC = Nursing Delirium-Screening Scale.

**Table 4:** The most common problems of patients—day 1 and 2.

**DAY 1**					
	**Medium**	**Median**	**Standard Deviation**	**Minimum**	**Maximum**
Disorientation	0.77	1.00	0.90	0.00	5.00
Behavior problems	0.49	0.00	0.73	0.00	3.00
Problems with communication	0.72	0.00	0.92	0.00	5.00
Illusions/hallucinations	0.62	0.00	0.83	0.00	4.00
Psychomotor delay	0.65	0.00	0.87	0.00	5.00
Result Day	1.00	1.00	0.96	0.00	6.00
Result Night	2.09	2.00	1.65	0.00	8.00
Result Other	0.17	0.00	0.79	0.00	6.00
**DAY 2**					
	**Medium**	**Median**	**Standard deviation**	**Minimum**	**Maximum**
Disorientation	0.62	0.00	0.73	0.00	3.00
Behavior problems	0.54	0.00	0.74	0.00	3.00
Problems with communication	0.65	0.00	0.84	0.00	4.00
Illusions/hallucinations	0.47	0.00	0.77	0.00	4.00
Psychomotor delay	0.55	0.00	0.80	0.00	4.00
Result Day	1.32	1.00	0.89	0.00	5.00
Result Night	1.28	1.00	1.13	0.00	6.00
Result Other	0.24	0.00	0.66	0.00	4.00

**Table 5:** Differences in the Illusion/Hallucination Scale and Day and Night scores.

	Day 1		Day 2		Statistics	
	Medium	Standard Deviation	Medium	Standard Deviation	*t*	*p*
Disorientation	0.77	0.90	0.62	0.73	1.783	0.076
Behavior problems	0.49	0.73	0.54	0.74	−0.831	0.407
Problems with communication	0.72	0.92	0.65	0.84	0.885	0.377
Illusions/hallucinations	0.62	0.83	0.47	0.77	1.984	0.049
Psychomotor delay	0.65	0.87	0.55	0.80	1.344	0.180
Result Day	1.00	0.96	1.32	0.89	−3.647	0.000
Result Night	2.09	1.65	1.28	1.13	5.839	0.000
Result Other	0.17	0.79	0.24	0.66	−0.935	0.351

t = student’s *t*-test; *p* = significance level.

## Data Availability

The authors declare that the data from this study are available from the authors S.K., D.O. and K.J. upon request.

## Supplementary Materials

The following are available online at https://www.mdpi.com/article/10.3390/ijerph181910108/s1, File S1: NuDesc scale interventions in 1st day of stay. File S2: Version of the Nursing Delirium Screening Scale (NuDESC). File S3: NuDesc scale interventions in 2nd day of stay.
