# Supplementary material for: The Polish Version of the Nursing Delirium Screening Scale (NuDESC PL)-Experience of Using in Nursing Practice in Cardiac Surgery Intensive Care Unit
Source: Int J Environ Res Public Health. 2021 Sep 26;18(19):10108. doi: 10.3390/ijerph181910108 (PMC8507661; doi:10.3390/ijerph181910108)
Supplement: Supplementary file 1 [file ijerph-18-10108-s001.zip › ijerph-1310945-SUPPLEMENTARY 2.pdf]

Nursing Delirium Screening Scale-NuDESC

Replaces Confusion Risk Screen and NEECHAM delirium screening tool on the Adult M/S flowsheet in Excellian

NURSING DELIRIUM SCREENING SCALE
Disorientation
Inappropriate Behavior
Inappropriate Communication
Illusions/Hallucinations
Psychomotor Retardation
NuDESC Score

Score NuDESC every shift, every day and if there is a *change in mentation* that occurs *anytime* during the shift.

Disorientation

0=Alert, oriented to person, place, time
1=Disoriented but easily reoriented
2=Disoriented x2 or x3 not easily oriented

Each cell contains 3 descriptors to choose from.

This is an observational screening tool. Please use your best judgment as to what the patient is demonstrating.

Inappropriate Behavior

0=Calm Cooperative
1=Restless and cooperative
2=Agitated pulling at devices climbing over side rails

Delirium can have fluctuating behaviors, one moment calm, and the other moment agitated. Please score tool again if behaviors change.

Inappropriate Communication

0=Appropriate
1=Unclear thinking or rambling speech
2=Incoherence, nonsensical or unintelligible speech

Use Family Caregiver Sheet if patient has cognitive impairment and is cared for by family members to give us insight to their needs.

Illusions/Hallucinations

0=None Noted
1=Paranoia, fears
2=Hallucinations, distortions of visual objects

Perceptual distortions accompanying delirium are usually visual.

Psychomotor Retardation

0=None
1=Delayed or slow responsiveness
2=Excessive sleeping, somnolent, lethargic

Delirium can be hypoactive, hyperactive or mixed. Be aware that hypoactive is the least detected by clinical staff.

NuDESC Score

Score > or = to 2 indicates patient is *screening* positive for delirium. Take action!

DELIRIUM INTERVENTIONS
Interventions if NuDESC score greater than or equal to 2:

Interventions if NuDESC score greater than or equal ...

Select Multiple Options: (F5)

- Promote nutrition: patient in chair for meals, has dentures, etc.
- Orient to current reality: (if does not increase agitation) modify envi
- Consult with the physician/CNS/NP/PA/Rx to discuss elimination c
- Pain management
- Discontinue bladder catheter as soon as appropriate
- Encourage mobilization
- Appropriate use of glasses and hearing aids
- Sleep promotion
- Monitor electrolytes
- Consider bladder scan to check for urinary retention
- If no BM in past 48 hours check for fecal impaction
- Any medications started or dose adjusted or stopped in past 24 ho
- Assess Vital signs and pulse oxygen
- Assess blood glucose
- Assess I&O signs of dehydration

Updated interventions for patients screening positive for delirium. Nursing interventions can make a difference is recognizing and treating delirium.
