# Supplementary material for: The Polish Version of the Nursing Delirium Screening Scale (NuDESC PL)-Experience of Using in Nursing Practice in Cardiac Surgery Intensive Care Unit
Source: Int J Environ Res Public Health. 2021 Sep 26;18(19):10108. doi: 10.3390/ijerph181910108 (PMC8507661; doi:10.3390/ijerph181910108)
Supplement: Supplementary file 1 [file ijerph-18-10108-s001.zip › ijerph-1310945-SUPPLEMENTARY 3.pdf]

Supplementary material 3.

NuDesc scale interventions in 2nd day of stay.

Interventions in 2nd day		N	%
Nutrition support - the patient eats meals while sitting on a chair, has an artificial jaw, etc.	Yes	202	100.0%
8 a.m.			
8 p.m.	No	202	100.0%
Summoning to reality (if that doesn't increase anxiety), changing surroundings	Yes	141	69.8%
8 a.m.	No	61	30.2%
8 p.m.	Yes	158	78.2%
	No	44	21.8%
Consultation of a doctor / clinical nurse) / registered nurse in the field of advanced nursing practice)	Yes	202	100.0%
8 a.m.			
8 p.m.	Yes	202	100.0%
Pain treatment	Yes	202	100.0%
8 a.m.			
8 p.m.	Yes	202	100.0%
Remove the catheter as early as possible	No	202	100.0%
8 a.m.			
8 p.m.	No	202	100.0%
Encourage movement	Yes	202	100.0%
8 a.m.			
8 p.m.	No	202	100.0%
Appropriate use of glasses and hearing aids	Yes	149	73.8%
8 a.m.	No	53	26.2%
8 p.m.	Yes	158	78.2%
	No	44	21.8%

Sleep aid	No	202	100.0%
8 a.m.			
8 p.m.	Yes	162	80.2%
	No	40	19.8%
Electrolyte monitoring	Yes	202	100.0%
8 a.m.			
8 p.m.	Yes	202	100.0%
Consider bladder ultrasound for urinary retention	No	202	100.0%
8 a.m.			
8 p.m.	No	202	100.0%
If there is no bowel movement in the last 48 hours, test for the presence of fecal stones	No	202	100.0%
8 a.m.			
8 p.m.	No	202	100.0%
All drugs introduced or withdrawn in the last 24 hours (or with a changed dose)	Yes	202	100.0%
8 a.m.			
8 p.m.	Yes	202	100.0%
Assessment of vital signs, pulse and saturation	Yes	202	100.0%
8 a.m.			
8 p.m.	Yes	202	100.0%
Testing the level of glucose in the blood	Yes	202	100.0%
8 a.m.			
8 p.m.	Yes	202	100.0%
Assessment of fluid balance in terms of dehydration	Yes	202	100.0%
8 a.m.			
8 p.m.	Yes	202	100.0%

N=number of patients
